# Cysticercal Encephalitis in a Young Female: A Rare Presentation of Neurocysticercosis

**DOI:** 10.7759/cureus.33931

**Published:** 2023-01-18

**Authors:** Nnenna E Ikeogu, Satyam Singh, Helai Hussaini, Zainab Omar, Sakshi Lakhra, Khalid H Mohamed, Munira Abdefatah Ali, Enoh Nguty Nkeng, Tulika Garg, Aadil Khan

**Affiliations:** 1 Internal Medicine, Abia State Faculty of Medicine, Abia, NGA; 2 Internal Medicine, Ganesh Shankar Vidyarthi Memorial Medical College, Kanpur, IND; 3 Otolaryngology, Liaquatian Research Council, Hyderabad, PAK; 4 Pediatrics, Dubai Medical College For Girls, Dubai, ARE; 5 Internal Medicine, All Saints School Of Medicine, St. Roseau, DMA; 6 Neurology, Sheffield Teaching Hospitals NHS Foundation Trust, Sheffield, GBR; 7 College of Medicine, University of Illinois at Chicago, Chicago , USA; 8 Public Health, DC Health, Washington, D.C., USA; 9 Medicine, Government Medical College and Hospital, Chandigarh, IND; 10 Department of Internal Medicine, Lala Lajpat Rai Hospital, Kanpur, IND

**Keywords:** albendazole, dress syndrome, tumour necrosis factor-alpha (tnfα), taenia solium, neurocysticercosis

## Abstract

One of the most frequent parasite infections of the central nervous system is neurocysticercosis. This neurologic condition is caused by *Taenia solium* (*T. solium*) larval infestation. Infected pork intake, poor hygiene practices, water tainted with *T. solium*, or asymptomatic carriers are the main ways of spread. We describe a case of neurocysticercosis in a young woman who presented with low-grade fever, headache, altered sensorium, and recurrent seizures. Computed tomography of the head revealed an inflammatory granuloma and a ring-increased attenuating lesion in the left temporal region. Additionally, a well-defined rounded discrete lesion was identified in the left parietal region on magnetic resonance imaging of the brain. Even if the symptoms do not initially suggest neurocysticercosis or if the patient lives in a region where the condition is uncommon, our case depicts adding neurocysticercosis to the differential diagnosis for encephalitis.

## Introduction

Neurocysticercosis (NCC) is an infection of the central nervous system (CNS) triggered by the ingestion of *Taenia solium* (*T. solium*) eggs. NCC has also been highlighted as a common etiology of community-acquired active epilepsy. A high percentage of lively epilepsy cases in developing international locations with India and Latin America [1,2]. A high prevalence of NCC was recorded among active epilepsy patients attending a tertiary care center in Assam, India. Ninety-three cases (61.2%) fulfilled the probable diagnostic criteria for NCC, which is greater than those reported by previous hospital-based studies from NCC endemic areas in South Africa (61.1%), Bhutan (25.4%), Rwanda (23.3%), Nepal (16.0%), Tanzania (2.0%-13.7%), Peru (12.0%), and rest of India including Odisha (43.7%), and Andhra Pradesh (27.5%) suggesting that NCC is a more significant public health problem in this region [3]. When humans eat pork contaminated with cysticerci, these develop into adult worms in the human intestine. However, humans can also become infected by internal and external autoinfection by ingesting the *Taenia solium* egg. After ingestion, tapeworm eggs hatch in the digestive system and release infectious oncospheres that traverse the intestinal lining and spread to all tissues via the bloodstream. After residing in the final location in the brain parenchyma, cysticerci remain asymptomatic over months or years before the initial symptoms appear [4,5]. Patients with NCC may be asymptomatic or manifest with nonspecific signs and symptoms such as cognitive decline, headache, focal deficit, or increased intracranial pressure. The clinical presentation depends on the number and location of lesions within the CNS and the degree of the host’s immune response to the organism [6]. Epidemiology, symptoms, signs, serology, and brain imaging are used to diagnose neurocysticercosis. Treatment includes using antiparasitic agents to treat viable cysts, usually accompanied by corticosteroids. Also, symptomatic treatment is given for seizures, inflammatory reactions, and hydrocephalus [7].

## Case presentation

A 24-year-old female was brought to the emergency department with complaints of altered mental status and recurrent seizures associated with low-grade fever, headache, and altered sensorium for the last three days. The seizure was acute in onset, generalized tonic-clonic in nature, and associated with tongue biting, blood-tinged frothing, and urinary incontinence. It was her second episode of abnormal body movement and altered sensorium after being admitted to the hospital. Each episode lasted for 20-30 minutes. She returned from a hill station a few days ago, and her family and social history were insignificant.

**Figure 1:**
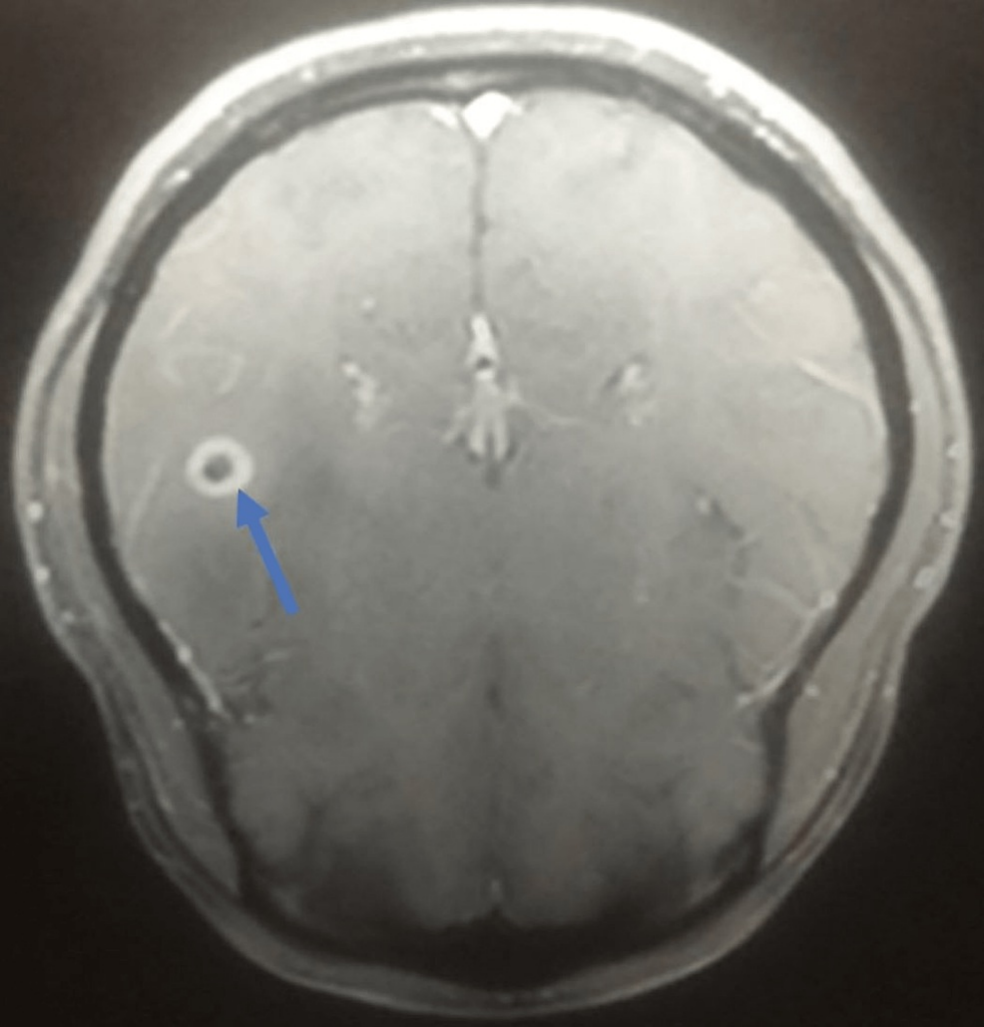
An inflammatory granuloma on the right temporal side.

**Figure 2:**
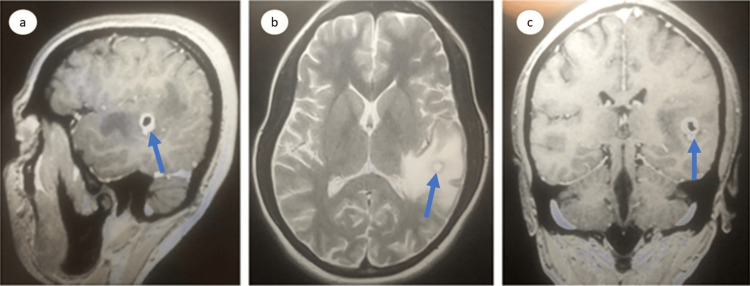
MRI brain with contrast revealing a well-defined rounded discrete lesion seen in the left parietal region as shown in the sagittal (a), trans-axial (b), and coronal (c) sections.

**Figure 3:**
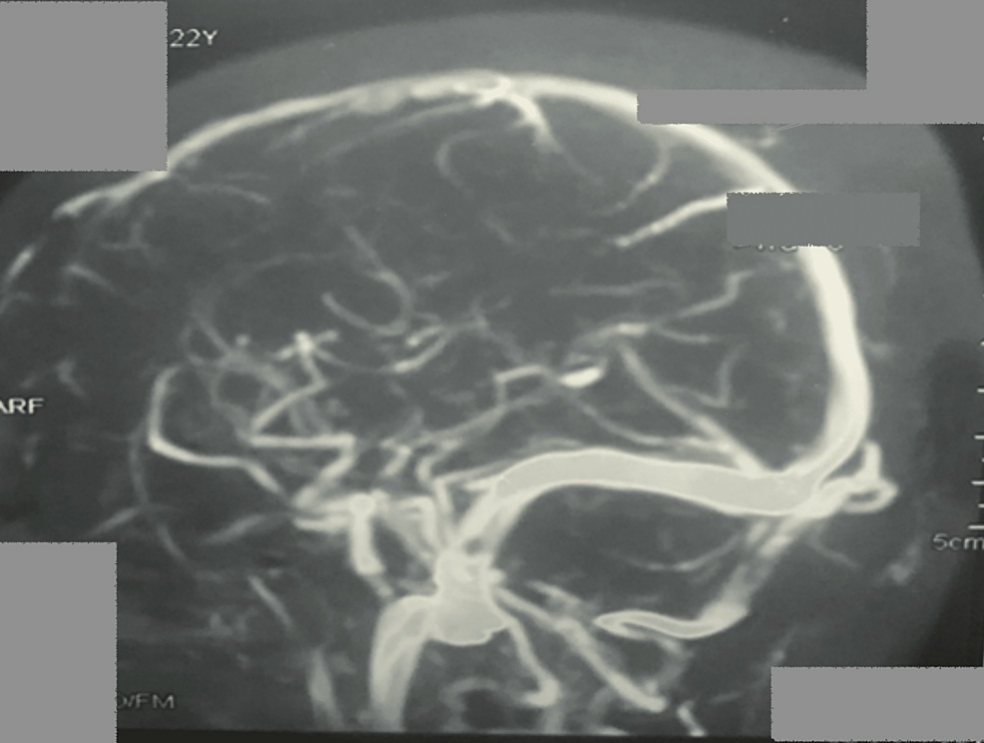
MR venogram revealing that the left transverse sinus is hypoplastic.

On admission, the patient was afebrile, with a blood pressure of 110/70 mmHg, a Spo2 of 97%, and a heart rate of 86/min. Her physical examination revealed a Glasgow coma scale (GCS) score of 11 (eye: 4, verbal: 2, motor: 5). Her rest systemic examination was unremarkable. Her initial laboratory investigations were within normal range except for mild elevation of transaminases (Table 1). Further evaluation with a non-contrast computed tomography (CT) head shows a ring-enhancing lesion in the left temporal region with perifocal edema, an eccentric, and an inflammatory granuloma (Figure 1). In addition, magnetic resonance imaging (MRI) brain showed a well-defined rounded discrete hyperintense lesion in the left parietal region with mild to moderate vasogenic edema, leading to partial effacement of the adjoining sub-arachnoid space and moderate peripheral rim enhancement (Figure 2). On follow-up after two weeks, an MR venogram revealed that the left transverse sinus was hypoplastic, as shown in Figure 3.

**Table 1:** Initial laboratory results.

Parameter	Laboratory value
Hemoglobin	12.9 (12-16.5) g/dL
Red blood cell count	4.1 (4.2-5.4) million cells/mcL
White blood cell count	11,200 (4,000-11,000)/mcL
Platelet count	141,000 (150,000-450,000)/mcL
Serum sodium	139 (135-147) mmol/L
Serum potassium	3.7 (3.5-5.1) mmol/L
Serum phosphorous	1.48 (1.12-1.45) mmol/L
Aspartate aminotransferase	78 (8-33) IU/L
Alanine aminotransferase	84 (34-40) IU/L
Lactate dehydrogenase	280 (105-333) IU/L

**Figure 4:**
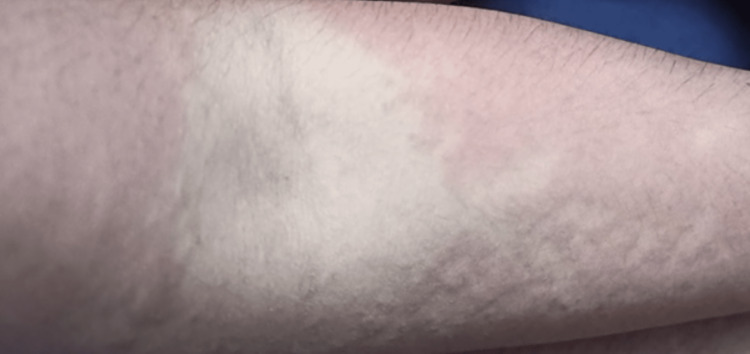
An urticarial, maculopapular eruption, and erythroderma as in arm.

Following confirmation of diagnosis, the patient was started on albendazole 400mg twice daily and carbamazepine. However, on follow-up after two weeks, the patient developed skin rashes, remarkably elevated liver enzymes, and total bilirubin (Figure 4).

After proper evaluation, immediately anti-epileptic drugs were discontinued, and for skin rashes, the patient was given oral fexofenadine and methylprednisolone, levetiracetam beside topical steroids. On subsequent follow-up, her rashes and deranged aminases resolved, and the patient was doing well.

## Discussion

Among CNS infections, NCC is considered the leading cause of acquired epilepsy in developing nations. Despite being deemed eradicable by the International Task Force for Disease Eradication of the World Health Organization in 1993, NCC is still recognized as a major neglected disease due to a lack of information about its burden and transmission, the lack of diagnostic tools available in resource-poor areas, and the lack of intervention strategies for its control [8]. Recent data indicate that NCC is a significant health problem in endemic regions, causing epilepsy in 0.6%-1.8% of the population in Latin American countries only [9].

Most patients with neurocysticercosis have few intracranial lesions. However, a small subset shows the development of massive infections that may be divided into encephalitic and non-encephalitic. Proper differentiation of both forms is significant because they have different pathogenetic mechanisms and require different therapeutic approaches. The encephalitic condition often occurs in children and young women without contact with the parasite until they are infected with a heavy load of *T. solium *eggs. In these cases, the host’s immune system actively reacts against the parasites. Intracranial hypertension occurs in patients with cysticercotic encephalitis, characterized by clouding of consciousness, seizures, diminution of visual acuity, headache, vomiting, and papilledema [5]. Other unusual manifestations of NCC are stroke, visual loss, ataxia, dystonia, dementia, and hydrocephalus [10]. Cysticercal granuloma has also been reported as a cause of Weber’s syndrome and ptosis. Our patient presented with features of raised intracranial pressure. He fulfilled the diagnostic criteria of neurocysticercosis [11]. Prasad et al. reported a case of cysticercal encephalitis with cortical blindness [12].

Cysts of NCC are most found in the brain parenchyma, followed by the meninges, ventricles, eyes, and spinal cord. The manifestations of NCC are diverse, and there are no specific signs and symptoms [5]. Acute symptomatic seizures with other clinical syndromes, such as dementia, focal neurological deficits, meningoencephalitis, severe headache, hydrocephalus, or psychological disorders, may be seen in a patient. In patients showing extra parenchymal NCC, the most common clinical symptom and sign is hydrocephalus [13]. The clinical presentation also depends on the stages and number of cysts. The treatment consisted of three types of drugs anti-parasitic for the cysticerci, anti-epileptic for the seizures, and corticosteroids for the inflammation. Corticosteroids are prescribed along with albendazole to reduce the severe inflammatory response. Imaging modalities of the brain, specifically CT and MRI, are crucial for diagnosing NCC [14]. Neuroimaging highlights more detailed information than immunodiagnostic tests, including the number, size, location, and stage of the parasites, as well as signs of any perilesional inflammation and associated conditions such as increased intracranial pressure, hydrocephalus, or other conditions. Immunodiagnostic and serological tests show eosinophilia, specific antibodies to the parasite, such as the lentil lectin glycoprotein enzyme-linked immunoelectrotransfer blot (LLGP-EITB) assay or circulating parasite antigens, thus supporting the etiological diagnosis. [15]

The use of the drug carbamazepine for the treatment resulted in the development of morbilliform rash over the body, which was associated with DRESS syndrome (drug reaction with eosinophilia and systemic symptoms) [16]. She also presented classical features of DRESS syndrome, including fever, rash, and lymphadenopathy, and systemic involvement of the liver was also seen in her. In this case, we tend to show the adverse effect of anti-epileptic drugs in treating patients suffering from neurocysticercosis. This study addresses a significant gap in the literature about the complications associated with using anti-epileptic drugs in patients with neurocysticercosis.

## Conclusions

NCC is considered the leading cause of acquired epilepsy in developing nations. Despite being deemed eradicable, NCC is still recognized as a major neglected disease due to the lack of information about its burden and transmission, the lack of diagnostic tools available in resource-poor areas, and the lack of intervention strategies for its control. Early recognition and management are crucial to prevent morbidity and mortality. Influence epilepsy distribution is essential to improve and tailor intervention programs and prevention strategies.
